# Treatment of Corns

**Published:** 1888-01

**Authors:** Lewis A. Sayre

**Affiliations:** Bellevue Hospital


### *TREATMENT OE CORNS.*

Clinic of Dr. LEWIS A. SAYRE, at Bellevue Hospital.

The first thing to do is to wear well-fitting shoes. With shoes sufficiently large to allow expansion of the foot in walking, you will have no corns; but, if any exist, it is necessary to remove them.

Begin by paring the corn, shaving off the epidermis with a sharp instrument, but without provoking bleeding. Afterwards, cauterize the surface with a crayon of nitrate of silver. In this way, you cause the elimination of the kernel, which you could not do with a bistoury without bleeding. Now smear the corn with diachylon; wrap a narrow bandage around, of sufficient thickness to prevent pressure.

If it be a soft corn, the best treatment is cauterization with nitric acid or nitrate of silver. After having removed, with a penknife or scissors, the superficial hard part of the corn, dry the surface, and apply caustic. The first cauterizations are generally painful, but the results are excellent.

After the cauterization is finished, insert between the toes tampons of cotton in a way so as to permit the circulation of air; at the expiration of several days, remove with forceps the eschar made by the caustic, and repeat the cauterization. The second cauterization is ordinarily painless, and rarely has to be repeated.

